# Changing outcomes of stem cell transplantation in primary immunodeficiencies: Results from a tertiary-care charitable trust hospital in Mumbai

**DOI:** 10.1016/j.jacig.2023.100105

**Published:** 2023-03-29

**Authors:** Ambreen Pandrowala, Mukesh Desai, Manisha Madkaikar, Shilpa Kulkarni, Lakshmi Shobhavat, Jayashree Mishra, Shreepal Jain, Parmarth Chandane, Kunal Sehgal, Saroj Chavan, Parag Karkera, Pradnya Bendre, Ameet Thanky, Sudha Rao, Shakuntala Prabhu, Minnie Bodhanwala, Bharat Agarwal, Prashant Hiwarkar

**Affiliations:** a Department of Blood and Marrow Transplantation, Bai Jerbai Wadia Hospital for Children, Mumbai, India; b Department of Inborn errors of Immunity, Bai Jerbai Wadia Hospital for Children, Mumbai, India; c Department of Paediatric Neurology, Bai Jerbai Wadia Hospital for Children, Mumbai, India; d Department of Intensive Care, Bai Jerbai Wadia Hospital for Children, Mumbai, India; e Department of Paediatric Cardiology, Bai Jerbai Wadia Hospital for Children, Mumbai, India; f Department of Paediatric Pulmonology, Bai Jerbai Wadia Hospital for Children, Mumbai, India; g Department of Paediatric Radiology, Bai Jerbai Wadia Hospital for Children, Mumbai, India; h Department of Paediatric Surgery, Bai Jerbai Wadia Hospital for Children, Mumbai, India; i Department of Physiotherapy, Bai Jerbai Wadia Hospital for Children, Mumbai, India; j Department of Paediatrics, Bai Jerbai Wadia Hospital for Children, Mumbai, India; k Department of Pediatric Immunology and Leukocyte Biology, ICMR–National Institute of Immunohaematology, KEM Hospital, Mumbai, India; l Sehgal Path Lab, Mumbai, India

**Keywords:** Primary immunodeficiency, stem cell transplant, primary immune regulatory disorders, outcomes, graft-versus-host disease, survival

## Abstract

**Background:**

Hematopoietic stem cell transplantation in primary immunodeficiency disorders has come a long way since the first transplant in 1968. In India, pediatric stem cell transplantation long-term survival outcomes range from 62.5% to 75%, compared to 90% in high-income countries.

**Objective:**

We present single-center data of primary immunodeficiency transplants with immune-reconstitution evaluation after transplantation from a charitable trust hospital.

**Methods:**

Retrospective data of children transplanted for primary immunodeficiency disorders from March 2019 to March 2022 in a newly established transplant unit were collected. Data of pretransplant infections and comorbidities, surveillance for carbapenem-resistant Enterobacteriaceae, transplant characteristics, donor source, graft-versus-host disease, posttransplant infections, immune reconstitution, overall survival at 1 year, and immunodeficiency-free survival were collated.

**Results:**

Twenty-one patients underwent transplantation for primary immunodeficiency disorders. The median age at transplantation was 3 years and 5 months (range, 7 months to 17 years). Seventy-five percent of the cohort had organ involvement, with lung being the most common organ involved, followed by central nervous system. Fifty-two percent of children had peritransplant infections, with most of them recognized at the pretransplant assessment. Among 20 of 21 children with engraftment, 94% had complete chimerism initially, with 33% developing mixed chimerism over time. The median duration of immunosuppression was 3 months after transplantation, and only 1 child required systemic graft-versus-host disease treatment for more than a year. Immune-reconstitution showed good T-cell recovery at 3 months and naive T-cell production at 6 months. There was no regimen-related or sepsis-related mortality. Overall survival of the cohort was 95% at 1-year follow-up. Immunodeficiency-free survival was 86% after a median follow-up of 20 months.

**Conclusions:**

Immunodeficiency-free and graft-versus-host disease–free survival can be achieved in the majority of children with primary immunodeficiencies using enhanced supportive care and the latest transplantation algorithms.

The first hematopoietic transplantation in patients with primary immunodeficiencies was reported in 1968, in 2 children with severe combined immunodeficiency and 1 child with Wiskott-Aldrich syndrome.^1^^,^^2^ Since then, significant progress has been made in the field of stem cell transplantation, while newer primary immunodeficiencies have been recognized.

In India, the first stem cell transplantation was performed in 1998 for a child with Wiskott-Aldrich syndrome.^3^ With advances in supportive care and access to generic drugs, transplant programs in India have reported better survival, but specialized services are currently largely restricted to private hospitals. Recently published outcomes in these centers show a 5-year overall survival rate of 68%, compared to 90% in high-income countries.^3^^,^^4^ Importing medications and newer technologies for graft manipulation has made transplantations more feasible but costly in this cohort of patients with comorbidities.

We present our data on primary immunodeficiency patients transplanted at a tertiary-care charitable hospital. To our knowledge, this is the first cohort from a non–private sector hospital in the country.

## Methods

We retrospectively evaluated data of children transplanted at our center for primary immunodeficiency from March 2019 to March 2022. All children had a genetic diagnosis of primary immunodeficiency, and functional assays were performed as required. Primary hemophagocytic lymphohistiocytosis (HLH) and Treg-opathies such as immune dysregulation, polyendocrinopathy, enteropathy, X-linked syndrome (IPEX) syndrome, LPS-responsive beige-like anchor (LRBA) deficiency, and cytotoxic T-lymphocyte–associated protein 4 (CTLA4) deficiency were subclassified as primary immunoregulatory syndromes.

Patient characteristics, presence of pretransplant comorbidities and organ involvement, conditioning regimen, infection, graft-versus-host disease (GvHD) prophylaxis, and transplant outcome were analyzed.

Infections occurring during the period of transplantation assessment until before initiation of conditioning were included as peritransplantation infections. For assessing the lung, chest computed tomography and pulmonary function test were performed at the time of transplantation assessment. If chest computed tomographic scan revealed any evidence of infection, bronchoalveolar lavage was performed and samples sent for multiplex PCR testing, including bacteria, viruses, and *Aspergillus,* Gram stain, geneXpert, galactomannan, bacterial, and mycobacteria growth indicator tube culture. Anasopharyngeal aspirate was done before conditioning chemotherapy to look for bacteria, viruses, mycoplasma, and *Pneumocystis carinii* by multiplex PCR. All children underwent weekly fecal surveillance for carbapenem-resistant Enterobacteriaceae (CRE), and CRE status guided the choice of first-line antibiotics for febrile neutropenia.

The definition of organ involvement for various organs was defined by site, as follows: (1) brain—any cerebrovascular accident or infection (1 child had cerebellar abscess); (2) gastrointestinal—any evidence of inflammatory bowel disease on gastrointestinal scope analysis or chronic parasitic or viral infection; (3) lung—any evidence of infection as per chest computed tomographic scan and bronchoalveolar lavage; and (4) skin—any recalcitrant dermatologic involvement such as eczema requiring immunosuppression, infections requiring ongoing antimicrobials, viral skin involvement in the form of multiple areas of molluscum, or multiple episodes of herpes zoster.

## Results

Twenty-one patients with primary immunodeficiencies underwent stem cell transplantation. Primary immunodeficiencies transplanted include severe combined immunodeficiencies, Wiskott-Aldrich syndrome, leukocyte adhesion deficiency (aka LAD), HLH, combined immunodeficiencies (deficiencies in hyper-IgM or dedicator of cytokinesis 8, aka DOCK8) and immune dysregulation (LRBA deficiency, CTLA4 deficiency, and IPEX syndrome). Patient characteristics are shown in Table I.

**Table I:** Patient characteristics

Patient no.	Age at transplantation (months)	Diagnosis	Weight percentile for age	Height percentile for age	Previous ICU stay	Organ involvement at time of HSCT
1	63	Griscelli syndrome with CNS HLH	10th-25th	<3rd	No	CNS, liver
2	56	IPEX	<1st	<1st	No	Kidney
3	42	WAS	<1st	3rd-50th	No	Lung
4	35	DOCK8 deficiency	3rd	1st-3rd	No	Lung
5	26	Hyper-IgM syndrome	3rd-50th	3rd-50th	Yes	No
6	7	SCID	<1st	<1st	Yes	Lung
7	147	DOCK8 deficiency	<3rd	<3rd	No	CNS
8	163	CTLA4 deficiency	<3rd	3rd-10th	No	GIT
9	49	Primary HLH with CNS involvement	3rd-50th	3rd-50th	Yes	CNS
10	100	Griscelli syndrome with CNS HLH	25th-50th	<3rd	No	No
11	83	LRBA deficiency	3rd-10th	10th-25th	No	Lung
12	12	SCID	3rd-10th	<1st	Yes	Lung
13	205	WAS	<3rd	<3rd	No	Lung
14	24	DOCK8 deficiency	50th	3rd-50th	No	Lung
15	39	DOCK8 deficiency	<1st	<1st	No	Lung
16	7	LAD type 1	<1st	1st-3rd	No	No
17	14	Griscelli syndrome with CNS HLH	3rd-50th	1st-3rd	No	No
18	13	SCID	<1st	50th-75th	Yes	Lung, spleen
19	112	Fanconi anemia	25th-50th	10th-25th	Yes	No
20	39	IBMFS-3	<1st	<1st	No	No
21	76	PAP	25th-50th	50th-75th	Yes	Lung

*CNS,* Central nervous system; *DOCK8,* dedicator of cytokinesis 8; *GIT,* gastrointestinal tract; *HSCT,* hematopoietic transplantation; *IBMFS-3,* inherited bone marrow failure syndrome 3; *ICU,* intensive care unit; *LAD,* leukocyte adhesion deficiency; *PAP*, pulmonary alveolar proteinosis; *SCID,* severe combined immunodeficiency; *WAS,* Wiskott-Aldrich syndrome.

**Fig 1:**
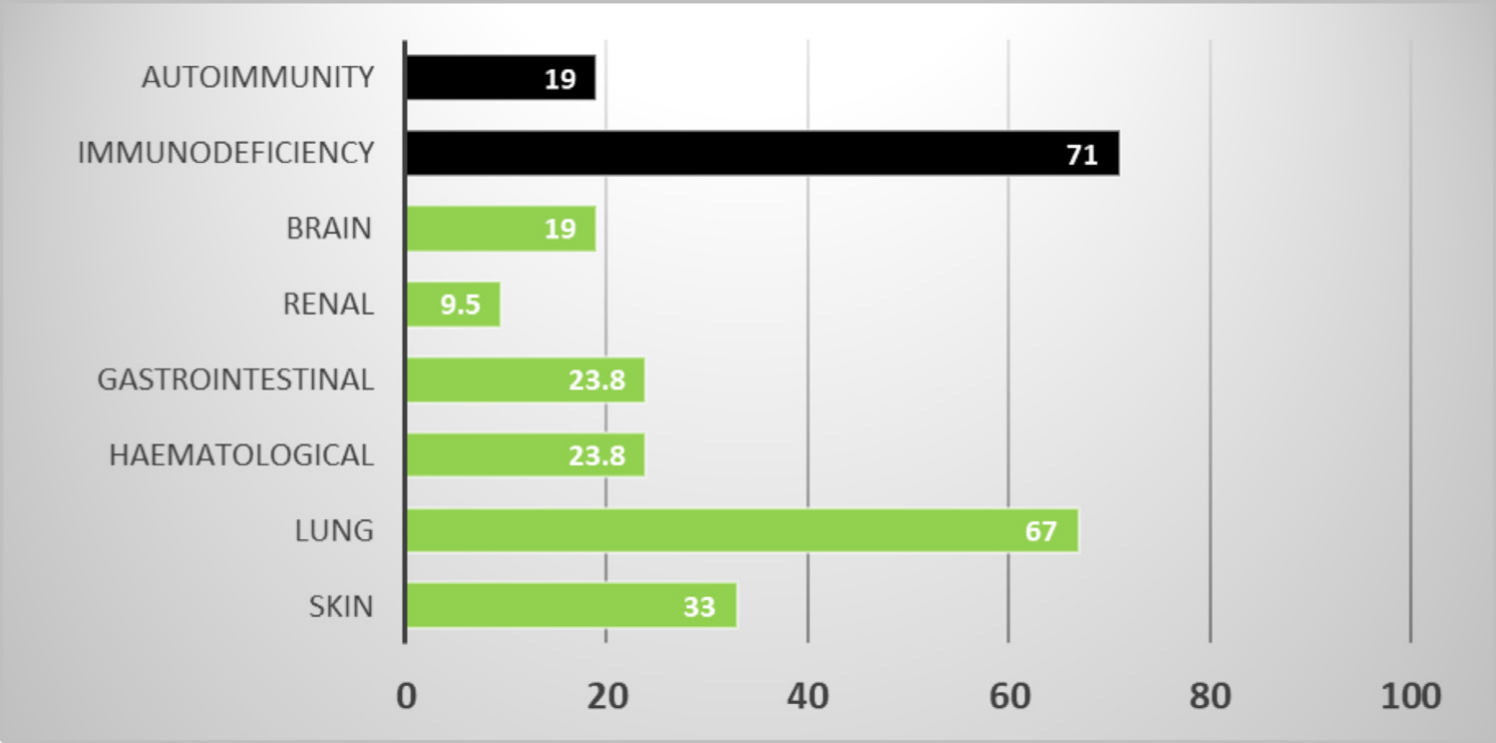
Clinical manifestations of 21 consecutive patients included in the study. Features of immune dysregulation such as autoimmunity and immunodeficiency are shown as *black bars;* organ involvement is shown as *green bars.*

The median age at transplantation was 3 years and 5 months (range, 7 months to 17 years). Fourteen (67%) of 21 children were of lower socioeconomic status as per the 2022 modified Kuppuswamy scale,^5^ and 7 (33%) of 21 were of middle socioeconomic status (median Kuppuswamy scale score of 8, range 4-25). Approximately 50% of the cohort had weight and height less than third centile for age at the time of transplantation, and one third weighed less than the first percentile, according to Indian Academy of Pediatrics growth charts. Seven of 21 children had a previous intensive care stay, with 3 children needing mechanical ventilation for acute respiratory distress syndrome. Three fourths of the cohort had some form of organ involvement, with lung being the most common organ involved, followed by skin and the gastrointestinal system (Fig 1).

**Fig 2:**
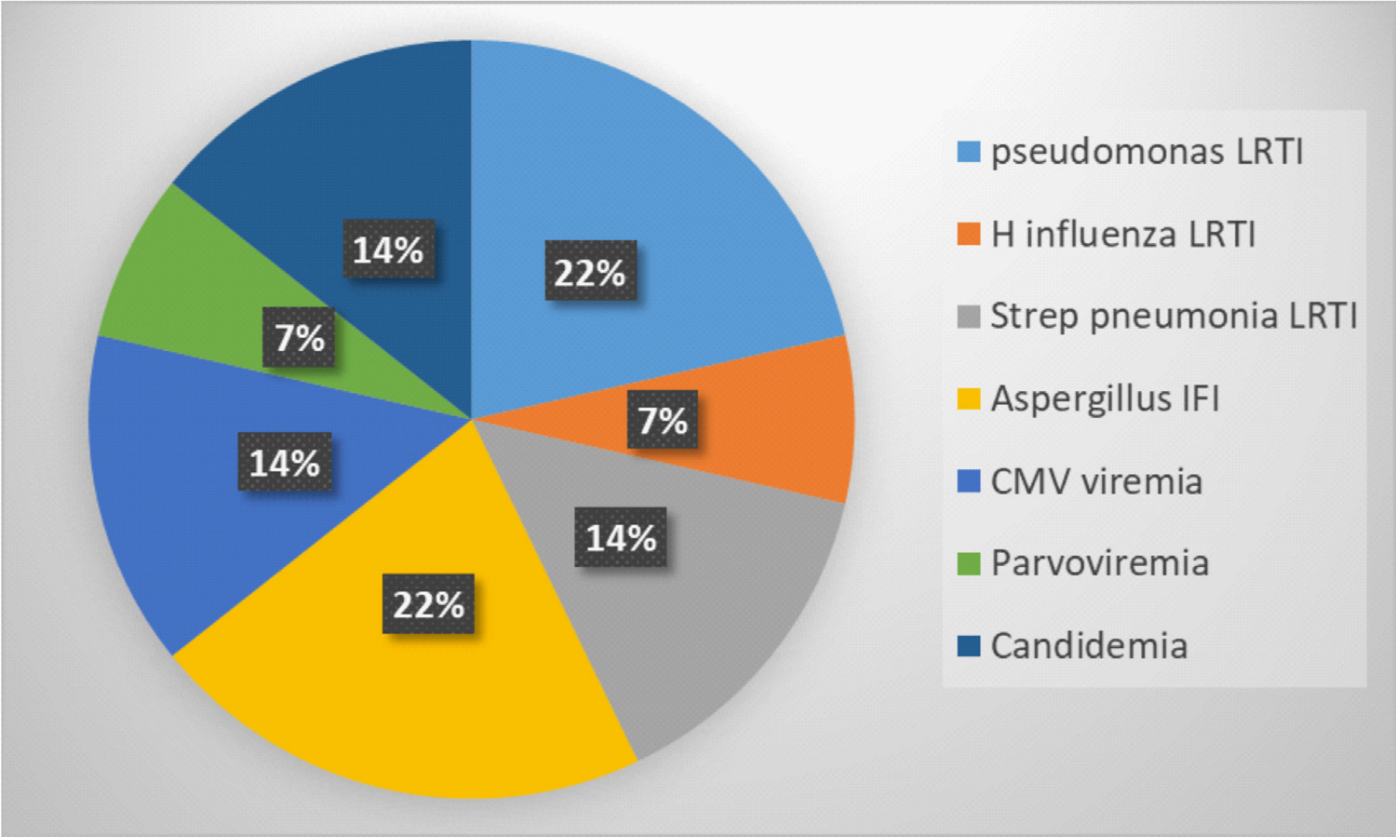
Infections detected at evaluation before transplantation.

Eleven (52%) of 21 children had peritransplant infections, with most recognized at pretransplant assessment. Occult infections diagnosed during pretransplant evaluation included viremia (cytomegalovirus [CMV] and parvovirus), candidemia, *Aspergillus* invasive fungal infection, *Haemophilus influenzae, Pseudomonas,* and *Streptococcus* pneumonia lower respiratory tract infections (Fig 2).

Of the 21 children in the cohort, 7 children (33%) had primary immunoregulatory disorders. Immune dysregulation in LRBA- and CTLA4-deficient children was managed with abatacept and sirolimus before transplantation. One child with IPEX syndrome was managed with steroids. All children with HLH (4 of 21) were assessed for central nervous system involvement and were managed according to the HLH 94 protocol before transplantation. Two children had ongoing HLH activity at the start of conditioning.

Donor source was matched family or sibling donor in 8 (38%) of 21 of cases, matched unrelated donor in 8 (38%) of 21, and half-matched parental in 4 (19%) of 21. One adopted child received a haploidentical matched unrelated donor transplant. Peripheral blood stem cells were used in all cases. Haploidentical grafts underwent TCR α/β depletion with or without CD45RA-depleted T-cell top-up. Alemtuzumab was provided as serotherapy in matched donor transplants (16 of 21), and rabbit anti-thymocyte globulin was provided in haploidentical transplants. Treosulfan–fludarabine–based reduced toxicity conditioning with thiotepa was provided in 14 (66%) of 21 of cases, or without thiotepa in 6 (29%) of 21 of cases. The child with Fanconi anemia received conditioning with fludarabine and cyclophosphamide. The median stem cell dose was 15 million CD34^+^ cells/kg (range, 6.7 million to 30 million CD34^+^ cells/kg). Median time to neutrophil engraftment was day +13 (range, 10-19 days), and platelet engraftment was day +11 (range, 9-33 days).

Two children had ciclosporin-induced pericardial effusion (10%), and 1 child had transplant-associated thrombotic microangiopathy, which resolved after stopping ciclosporin. No toxicity related to conditioning regimen was seen. The child with LRBA deficiency had periengraftment syndrome with a capillary leak, which was managed with defibrotide and abatacept. Engraftment was seen in 20 (95%) of 21 cases. One child with primary HLH died of severe cytokine release syndrome during the periengraftment period. Among 20 children who experienced engraftment, 94% had complete chimerism initially, with 33% developing mixed chimerism over time. No donor lymphocyte infusions were provided.

There were 3 culture-positive bloodstream infections in the cohort over a period of 3 years. One episode of bacteremia with fully sensitive *Pseudomonas* and 2 episodes of candidemia occurred during transplantation. Sixty-seven percent of children had positive CRE screening results but no CRE bacteremia was seen.

Forty-seven percent of children had CMV viremia (10 of 21) and were treated preemptively. Of 10 children with CMV viremia, 3 had pretransplantation CMV, with 1 having CMV pneumonia requiring mechanical ventilation. No CMV disease was seen after transplantation. Two children developed adenoviremia (9.5%). No Epstein-Barr viremia was seen.

The incidence of acute GvHD was 19%. Two children had only skin GvHD (grade 1), and 2 children had skin with gut GvHD (grade 2 and grade 3). No liver GvHD was seen. One child had limited skin chronic GvHD. The median time to stop GvHD prophylaxis was 3 months after transplantation, and only 1 child required systemic GvHD treatment for more than a year.

**Fig 3:**
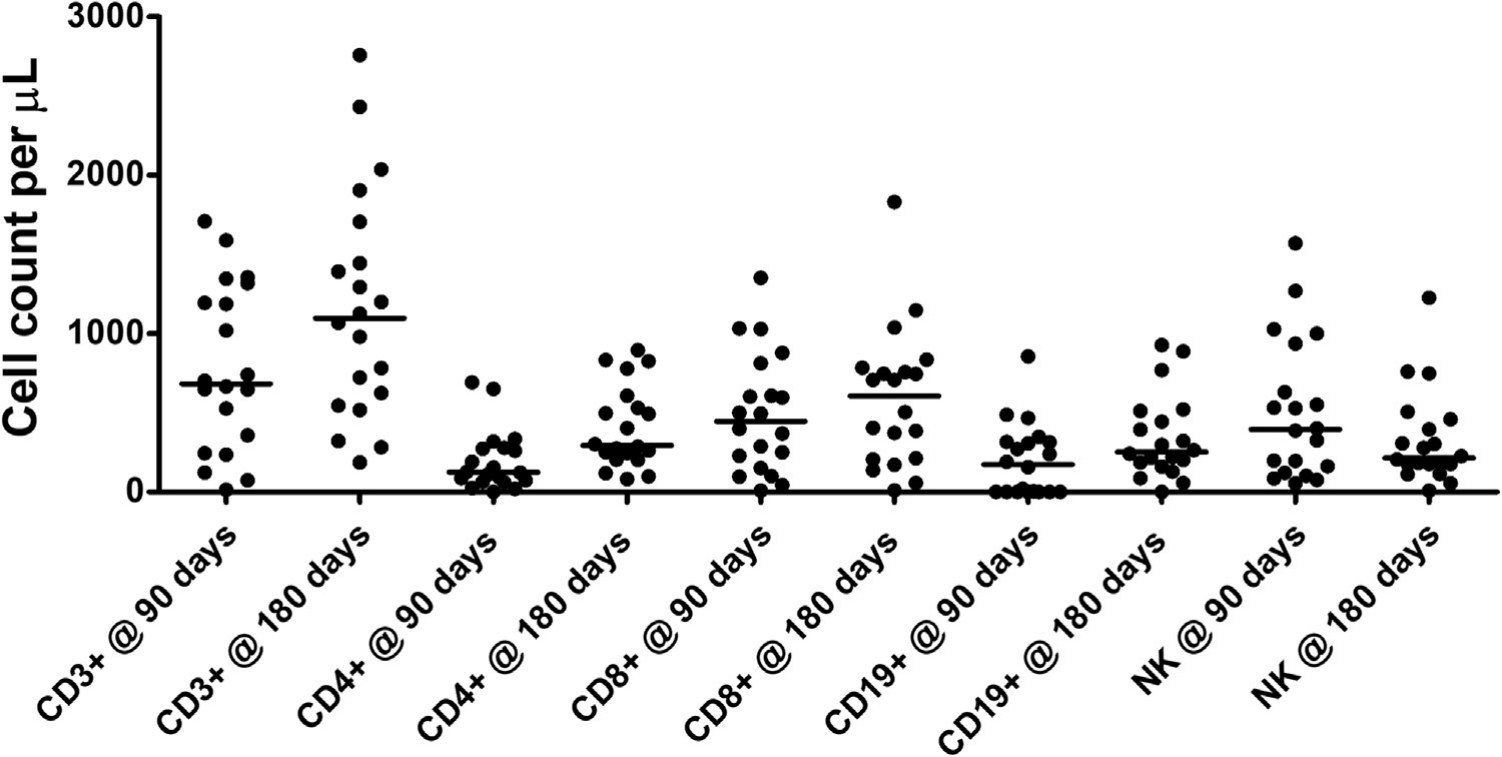
CD3^+^ T cell, CD4^+^ T cell, CD8^+^ T cell, B cell, and natural killer (NK) cell reconstitution at day +90 and day +180 after transplantation. Early recovery of T cells was observed.

Immune reconstitution assessed 3 months after transplantation showed good T-cell recovery, with a median (range) T-cell count of 684 (14-1710) cells/μL, a CD4^+^ T-cell count of 125 (1-692) cells/μL, and a CD8^+^ T-cell count of 447 (9-1352) cells/μL. Assessment at 6 months showed naive T-cell production, with a median (range) CD3^+^ T-cell count of 1164 (187-2758) cells/μL and CD4^+^ T-cell count of 352 (82-896) cells/μL, CD8^+^ T-cell count of 606 cells/μL with a median CD19^+^ cell count of 253 cells/μL, and natural killer cell count of 216 cells cells/μL. Median naive CD4 was 23.95% (range, 4.9-63%) of CD4^+^ T-cell compartment. CD3^+^ T-cell count, CD4^+^ T-cell count, CD8^+^ T-cell count, CD19^+^ T-cell count, and NK cell count are shown in Fig 3.

**Fig 4:**
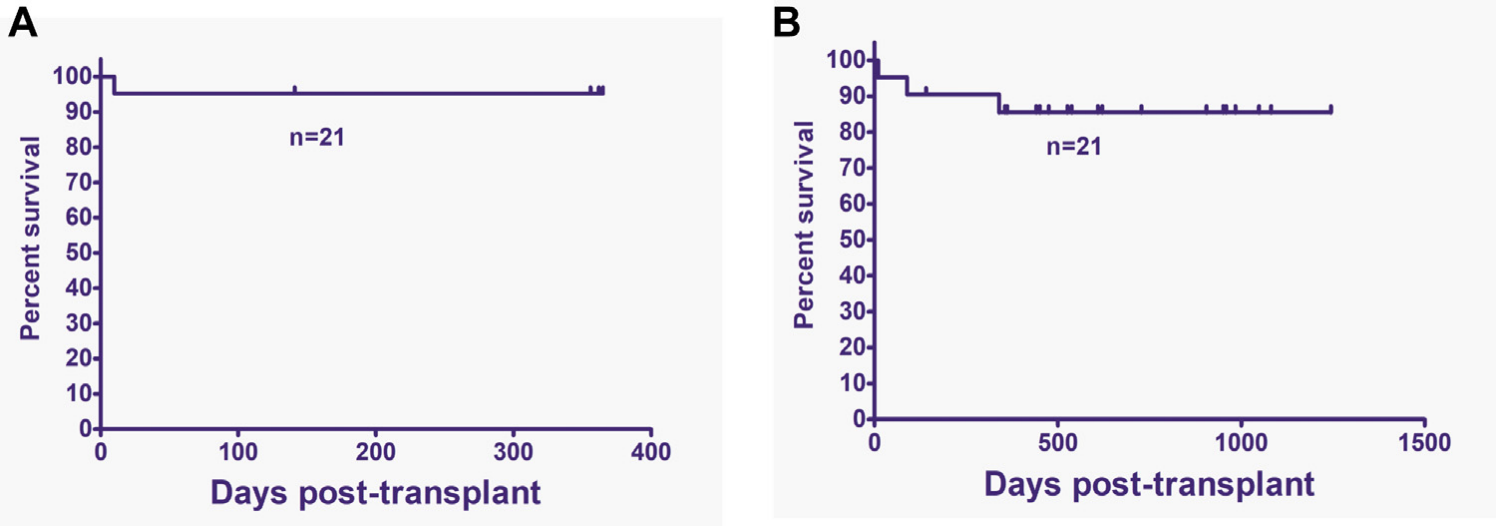
**(A)** Overall survival at 1 year after transplantation. **(B)** Disease-free survival at median follow-up of 20 months.

The cohort’s overall survival was 95% at 1 year of follow-up (Fig 4, *A*). Immunodeficiency-free survival was 86% after a median follow-up of 20 months in the entire cohort (Fig 4, *B*). All 14 children with primary immunodeficiencies without immune dysregulation are free of disease. Two children with immunoregulatory disorders (CTLA4 deficiency and IPEX syndrome) experienced disease relapse and required prolonged immunosuppressive therapy. The patient with CTLA4 deficiency had ongoing gut inflammation despite full donor chimerism, and the patient with IPEX syndrome had mixed chimerism and relapse of autoimmunity.

## Discussion

Previously published literature on stem cell transplantation in primary immunodeficiency disorders from India and other countries from Latin America and Middle East is shown in Table E1 in the Online Repository at www.jaci-global.org.^10^, ^11^, ^12^, ^6^, ^7^, ^8^, ^9^ Overall survival in the studies reported from India has ranged from 50% to 75%, and GvHD incidence from 33% to 50%. Immune-reconstitution data are unavailable in most published studies. One multicenter study of 228 patients reported an overall survival of 68%.^3^ Data on GvHD were unavailable in this study. Uppuluri et al published a single-center experience of 16 patients who underwent haploidentical transplantation with posttransplantation cyclophosphamide; overall survival was 62.5%, and there was a high GvHD incidence of 50%.^10^

Reports of 2 of the earliest cohorts were published in 2018. Uppuluri et al published a retrospective series of 85 patients with primary immunodeficiencies receiving a transplant at a single center with an overall survival of 67% and a GvHD incidence of 39%.^7^ In the cohort by Rastogi et al, cyclophosphamide was prescribed after transplantation, along with fludarabine, cyclophosphamide, and total body irradiation with or without thiotepa as the most common conditioning regimen.^6^ Two children in the cohort died before engraftment, of sepsis and transplant-associated thrombotic microangiopathy, for an overall survival of 75%.

In the multicenter cohort, data were reported from 7 centers across the country, all private sector hospitals.^3^ Two hundred twenty-eight children were reported to have undergone transplantation from 1998 to 2019, with 32% mortality and median follow-up of 14.4 months. A total of 18.9% of children underwent matched unrelated donor transplantation. The data do not cover GvHD incidence or immune reconstitution, but they showed infection as the leading cause of mortality at 53.9%, followed by GvHD at 17%, graft rejection at 10.6%, and regimen-related toxicity at 13.1%. The 5-year overall survival of the cohort was 68%, with poor quality of life in 15% of survivors as a result of chronic GvHD, poor immune reconstitution, and graft rejection.

Children with primary immunodeficiency disorders need a comprehensive pretransplantation evaluation to assess and identify any occult or partially treated infections and organ involvement. This helps in treating infections appropriately and individualizing the conditioning regimen to reduce the incidence of regimen-related toxicity. We found no incidence of regimen-related toxicity resulting in death.

Multidrug-resistant bacteria have become more prevalent since 2012 in India.^3^ In the published literature from India and upper-middle-income countries, sepsis and infection were the leading causes of mortality.^3^^,^^6^, ^7^, ^8^, ^9^, ^10^, ^11^, ^12^, ^13^, ^14^, ^15^, ^16^, ^17^ In our cohort, 67% of children had CRE colonization on surveillance screening, and 3 (14%) of 21 had *Pseudomonas aeruginosa* in bronchoalveolar lavage. None of the children died of infection. Early initiation of appropriate antibiotics based on weekly fecal surveillance data, as well as frequent monitoring of sepsis parameters such as procalcitonin, along with timely supportive-care interventions, could be among the reasons we did not observe mortality due to infections.

In the study cohort, we observed immunodeficiency-free and GvHD-free outcomes comparable to the outcomes from the best international centers. The outcomes were observed despite severe failure to thrive in half of the patients, previous intensive care admission in one third of the patients, and lower socioeconomic status in two thirds of the patients. The good outcomes observed in our cohort were because of the following: (1) the various strategies we used to identify pretransplantation infection and our aggressive infection treatments, (2) the preemptive provision of antibiotics according to CRE status, (3) careful donor selection, and (4) provision of alemtuzumab in HLA-matched grafts.

We observed negative outcomes such disease recurrence and treatment-related mortality in primary immunoregulatory disorders. Primary immunoregulatory disorders are difficult to treat. A relatively low survival rate was reported in a multicenter international study.^18^ Multiorgan involvement before transplantation and poor control of immune dysregulation are likely to be the main determinants of survival in this patient group.^19^ Therefore, it is crucial to establish the diagnosis early and consider transplantation before multiple organs are involved.

Primary immunodeficiency care has a long road ahead in India, with early diagnosis and timely referral being central to improving outcomes. Ours is the first cohort from India to report immune reconstitution in children with primary immunodeficiencies undergoing stem cell transplantation. Lymphocyte subset analysis is an important tool for guiding monitoring for viral reactivations and prediction of survival.^20^^,^^21^ Assessment at 3 months in the study cohort showed good T-cell count; assessment at 6 months showed naive T-cell output from the thymus.

We present the first transplantation cohort from a non–private sector hospital with overall survival of 95% at 1 year and immunodeficiency-free survival of 86% at a median follow-up of 20 months. The application of enhanced supportive care and the latest transplantation algorithms could improve outcomes in children with primary immunodeficiencies globally.

**Key messages**

• Immunodeficiency-free and GvHD-free survival can be achieved globally in most children with primary immunodeficiencies.
• Application of enhanced supportive care and the latest transplantation algorithms could improve outcomes in children with primary immunodeficiencies.
