# Supplementary material for: Changing outcomes of stem cell transplantation in primary immunodeficiencies: Results from a tertiary-care charitable trust hospital in Mumbai
Source: J Allergy Clin Immunol Glob. 2023 Mar 29;2(3):100105. doi: 10.1016/j.jacig.2023.100105 (PMC10509861; doi:10.1016/j.jacig.2023.100105)
Supplement: Supplementary Table S1 [file mmc1.pdf]

Author	Year	Cohort	Disease type	No of pts	Donor type	Serotherapy	Type of haplo transplant	Deaths due to bacterial infections	GvHD incidence	BCG reactivation	Graft failure	Overall survival	Median follow up
Rastogi et al [6]	2018	Single centre	SCID<br>HLH<br>WAS	8	All haplo	62% rATG<br>37% Alem	PtCy	1 sepsis death	33%- acute GvHD<br>25%- chronic GvHD	1/8	0%	75%	25 months
Uppuluri et al [7]	2018	Single centre	SCID 34%<br>WAS 17%<br>HLH 16%	85	44% MRD<br>9.5% MUD<br>16% cord<br>29% haplo	ATG- all MUD	4% CD34 selection and Alem in bag<br>8% CD3/19 selection<br>24% TCRa/b depletion<br>60% PtCy	4 sepsis death	39%<br>GvHD- cause of death in 12%	NA	5.8%	67%	NA
Aluri et al [8]	2019	Multicentric	SCID	4	25% MSD<br>50% haplo<br>25% cord	50% ATG	NA	1 sepsis death	1 GVHD death	NA	-	All expired	-
Gupta et al [9]	2019	Single centre	Griscelli syndrome	2	1-MRD<br>1-cord	NA	-	1 sepsis death	No	NA	0%	50%	24 months
Uppuluri et al [10]	2019	Single centre	5 WAS<br>3 HLH<br>2 Griscelli<br>2 MSMD<br>1- CHS, ORAI-1, SCID, Hyper IgM	16	All haplo	Not given	PtCy	4 sepsis death	50%- acute GvHD,<br>12.5%- chronic GvHD	NA	25%	62.5%	23.3 months
Raj et al [3]	2020	Multicentric	SCID 25%<br>HLH 25%	228	36.8% MRD<br>44% haplo<br>18.9% MUD	NA	57% PtCy<br>43% TCRalphabeta	53.9% of deaths due to infections	17% of death due to GvHD	NA	10.6% of deaths due to graft rejection	68%	14.4 months
Paul et al [11]	2021	Single centre	HLH	10	20% MRD<br>40% MUD<br>40% Haplo	NA	NA	2 sepsis death	1 death due to GvHD	NA	NA	60%	NA
Suri et al [12]	2021	Multicentric	WAS	25	20%MRD<br>16% MUD<br>40% haplo<br>20% cord	40% ATG	NA	4 sepsis death	41% (2 GvHD deaths)	NA	8%<br>1 death due to graft failure	64%	NA
Amayiri (Jordan)[13]	2013	Single centre	SCID<br>WAS<br>HLH	28	61% MRD<br>28.5%haplo<br>10.5% cord	14% Alem	Unmanipulated maternal	21% ICU stay for sepsis	57% acute<br>32% chronic	32%	31%	72%	32 months
Haskologlu (Turkey) [14]	2018	Single centre	High risk PID	15	66% MRD<br>20% MUD<br>13% haplo	13% ATG<br>6% alem	Both CD3+/CD19+deplete	NA	53% acute<br>20% chronic	33%	13%<br>1 primary, 1secondary	86.7%	32 months
Fernandes et al (Brazil) [15]	2018	Multicentric	SCID<br>WAS<br>Other PID	221	20%MRD<br>29%MUD<br>37%MMUD<br>12%haplo	62% rATG<br>7.6% Alem	22 T deplete<br>13 PtCy	55% deaths (35/64)	23%- acute GvHD<br>12%- chronic GvHD<br>(6% extensive)	28/53 vaccinated patients developed	12% primary<br>9% secondary	71.6%	57 months

										disseminated BCG			
Cipe (Turkey) [16]	2021	Single centre	SCID<br>WAS<br>HLH<br>DOCK8	58	41% MRD<br>34% MUD<br>24% haplo	rATG- all except MSD	PtCy in all haplo +3MUD	7/15 deaths due to sepsis	24% acute,<br>10% chronic	56% (one death)	1.7%	74%	56 months
Staines-Boone (Mexico) [17]	2022	Multicentric	CID<br>Phagocytic<br>Others	19	37% cord<br>63% haplo	NA	PtCy	NA	21% GvHD	NA	NA	73%	NA
Our data	2022	Single centre	SCID<br>WAS<br>HLH<br>Hyper IgM,<br>DOCK8 deficiency<br>LRBA,<br>CTLA4,<br>IBMFS-3,<br>Fanconi,<br>CSF3R	21	38% MRD<br>38% MUD<br>19% Haplo	76% Alem for HLA-matched donors<br>24% rATG for haploidentical donors	All TCR alpha-beta depletion	no deaths	19% acute<br>4% chronic	14%	0%	95%	15.3 months

Table E1 Published literature on stem cell transplant in primary immunodeficiency disorders in India, Jordan, Turkey, Brazil, and Mexico.
NA - not available, Alem - Alemtuzumab, MRD - Matched related donor, MUD - Matched Unrelated Donor, haplo-haploidentical donor, ATG - Anti-thymocyte globulin, PtCy - Post transplant cyclophosphamide
